# Comparison of mouse ovarian follicular development and gene expression in the presence of ovarian tissue extract and sodium selenite: An experimental study

**DOI:** 10.18502/ijrm.v21i5.13476

**Published:** 2023-05-12

**Authors:** Hamed Shoorei, Mina Jafarabadi, Shahram PourBayranvand, Mojdeh Salehnia

**Affiliations:** ^1^Department of Anatomy, Faculty of Medical Sciences, Tarbiat Modares University, Tehran, Iran.; ^2^Department of Anatomical Sciences, Faculty of Medicine, Birjand University of Medical Sciences, Birjand, Iran.; ^3^Vali-E-Asr Reproductive Health Research Center, Family Health Research Institute, Tehran University of Medical Sciences, Tehran, Iran.

**Keywords:** Follicle-stimulating hormone receptor, Ovary, Sodium selenite, Proliferation cell nuclear antigen, Mouse.

## Abstract

**Background:**

Ovarian tissue extract (OTE) and sodium selenite (SS) enhance the growth and maturation of preantral follicles in a dose-dependent manner.

**Objective:**

The present study was designed to bring more information regarding the mechanism of OTE and SS on the mRNA expression of follicle-stimulating hormone receptors (FSHR) and the proliferation cell nuclear antigens (PCNA) of in vitro matured isolated follicles.

**Materials and Methods:**

The tissue extract was prepared from adult ovaries. The preantral follicles (n = 266) were isolated from 12-16-day-old mice and cultured in the control, experimental I (10 ng/ml SS), and experimental II (OTE) groups for 12 days. The follicular diameter, survival, and maturation rates, also, the production of 17-β-estradiol and progesterone, and the follicular expression of *PCNA* and *FSH *receptor genes were analyzed.

**Results:**

The survival rate of follicles in the SS-treated group (84.58%) was significantly higher than that OTE (75.63%; p = 0.023) and control (69.38%; p = 0.032) groups. The mean diameter of culture follicles in experimental group I (403.8 μm) and experimental group II (383.97 μm) increased significantly in comparison with the control group (342.05 μm; p = 0.032). The developmental rate of follicles, percentages of antrum formation, released metaphase II oocytes (p = 0.027; p = 0.019 respectively), production of hormones and the expression of 2 studied genes were significantly increased in both experimental groups in compare with control group (p = 0.021; p = 0.023 respectively).

**Conclusion:**

The OTE and SS have a positive effect on development of mouse preantral follicles via over-expression of *FSHR* and *PCNA* genes.

## 1. Introduction 

Folliculogenesis in mammals is one of the most complex processes that involves direct interaction between somatic cells (such as granulosa and theca cells) and oocytes (1). Different factors such as hormones, growth factors, and antioxidants play critical roles in the growth and maturation of follicles and oocytes (2). Several culture systems, including 2- and 3-dimensional systems, were designed to investigate the effects of these factors during in vitro follicular culture. Culture media usually contain necessary supplementations such as serum, growth factors, hormones, and so on that improve follicular maturation (3).

It has been reported that selenium salts as non-metal chemical elements in large amounts are very toxic, but trace amounts of selenium are necessary for animal cell function (4). The addition of selenium to the culture medium as an antioxidant leads to the maturation of follicles and oocytes (5-10). It was reported that the supplementation of culture media with sodium selenite (SS) could increase the growth and maturation of preantral follicles (5). Also, several studies have demonstrated that supplementation of mouse oocyte maturation media with SS improved not only the oocyte developmental rate but also enhanced its expression of mitochondrial transcription factor A and mitochondrial DNA (6-9). These studies have shown that the reactive oxygen species (ROS) level was decreased in the presence of SS, which acts as an antioxidant (5, 7). It was also shown that selenium is one of the main critical factors in follicular fluid (11).

Ovarian follicular development is also regulated by intra-ovarian factors such as steroid hormones, epidermal growth factor, vascular growth factor, fibroblast growth factor, and growth differentiation factor β that enhance follicular development (11, 12). Some of these factors are produced by the ovarian stromal cells and follicular cells (12). The ovarian microenvironment could improve follicular development by providing these elements. However, the literature shows that supplementation of culture media with follicular fluid could enhance the development and the quality of cumulus-oocyte complex and embryo (12, 13).

Previously, we studied the effects of different concentrations of ovarian tissue extract (OTE) on mouse follicular development. The OTE contains components that mimic the natural composition of ovarian tissues and was obtained from adult mouse ovaries, which contain a lot of follicular fluid that has an impact on follicular development. Our results at the morphological level demonstrated that the OTE increased the development of follicles as its concentration increased within the culture medium (14).

Moreover, this study was designed to bring some additional information and compare the effect of SS and OTE on follicular function and development. In addition, at the mRNA level, the expression of 2 critical genes including follicle-stimulating hormone receptor (FSHR) and proliferation cell nuclear antigen (PCNA) that are involved in the maturation and proliferation of were assessed.

## 2. Materials and Methods

The reagents were prepared by Sigma Aldrich company (Dusseldorf, Germany) except those indicated in the text.

### Study design 

In this experimental study from mouse ovaries, the follicles were dissected with 145-155 μm diameter and divided into 3 groups as follows. In the control group the culture media (α-minimum essential medium; αMEM) containing 1% insulin, transferrin, and selenium (Invitrogen, Paisley, UK), 100 mIU/ml recombinant follicle-stimulating hormone (rFSH or Gonal-f; Serono, Switzerland), 100 IU/ml penicillin and 100 mg/ml streptomycin and 10% fetal calf serum (FBS; Gibco, UK). In experimental group I, the culture media was the same as the control, in addition, 10 ng/ml SS was added (5). In experimental group II, the described culture media was used and the FBS was replaced with OTE (14). After 12 days of culture, the survival and developmental rate of follicles and the expression of *FSHR* and *PCNA* genes were analyzed and compared. In addition, the levels of 17-β-estradiol and progesterone in the collected media were analyzed.

### Animals and collection of ovarian tissue

For this study, 12-16-day-old female NMRI mice (n = 20) ovaries that contain preantral follicles were used for follicular isolation and 6-8-wk-old adult mice (n = 15) for the preparation of OTE. The mice were maintained in the animal house of the university under standard conditions (12-hr light/12-hr dark cycle, 22-24 C, and 55% humidity). The ovaries were obtained after mice cervical dislocation, then transferred to culture media consisting of 10% FBS, 100 IU/ml penicillin, and 100 mg/ml streptomycin until assessments.

### OTE preparation

The OTE was prepared according to the previous study (14). After dissecting the adult mice ovaries under a stereomicroscope, they (n = 15) were collected and immersed in potassium ammonium chloride solution (pH = 7.2-7.4) for 1 min. Ovaries were then washed 3 times with phosphate-buffered saline (PBS) and put into a glass homogenizer tube. They were homogenized by adding 2 ml of Tris-HCL buffer (pH = 7) and 50 μL of protease inhibitor solution. Then the volume of the solution reached 10 ml after the homogenization of ovarian tissues by Tris-HCL buffer (pH = 7). The obtained solution was sonicated for 2 min under W50 sonication conditions. Finally, the solution was centrifuged at 12000 RPM for 15 min. The supernatant was collected as tissue extract and stored at -80 C until assay.

### Total protein analysis

500 μL of supernatant of OTE was used to measure the amount of protein. The amount of protein in fetal bovine serum (FBS) was used as a reference to select the concentration of OTE. According to our previous study, the OTE which contains ½ of the protein level in FBS was selected (14).

### 3-dimensional follicle culture

A 3-dimensional culture system based on sodium alginate capsulation was used for follicular culture (14). A mixture of sodium alginate and activated charcoal in distilled water was prepared to eliminate organic pollution. After 24 hr it was diluted with PBS at a concentration of 0.5% (w/v), then each isolated follicle was individually transferred into 7 µL of sodium alginate and a cross-linking solution containing 50 mM CaCl ${}_{2}$ and 140 mM NaCl, respectively. After washing the follicles in PBS at least 2 times for 2 min in each, the capsulated follicles (n = 248 in total) were put in the medium under mineral oil. The group without SS and OTE was considered as the control group (n = 90) and in this group, the αMEM culture media was supplemented with 1% insulin, transferrin, and selenium, 100 mIU/ml rFSH, 100 IU/ml penicillin, and 100 mg/ml streptomycin and 10% f FBS. In experimental group I (n = 86 follicles) the FBS was replaced by 10 ng/ml SS (5) and in experimental group II (n = 90 follicles) with OTE as its protein concentration was ½ of FBS protein (14). The half of culture media was renewed by fresh ones at one-day intervals during the culture period and the remaining media was frozen until analysis.

### In vitro ovulation induction

The morphological changes of follicles were studied using inverted microscope, and the follicles that showed a dark appearance were considered dead. By supplementing the culture media with 1.5 IU/ml of human chorionic gonadotropin (hCG; Organon) the oocyte ovulation induction was induced on Day 12 of culture, then 18 hr later the oocyte staging was done. The germinal vesicle oocyte has the prominent nucleus that is arrested in the prophase of meiosis I; the nucleus and polar body were not visible in the germinal vesicle breakdown oocyte, and the presence of the first polar body was demonstrated in the metaphase II (MII) oocyte (13).

### Hormonal assays

At the end of the culture period, steroid hormones, including 17-β-estradiol and progesterone, were analyzed in the collected media (n = 3 in each group). The levels of 17-β-estradiol and progesterone (Biotest AG, Dreieich, Germany) were measured by an enzyme immunoassay, and these experiments were done in triplicates.

### RNA extraction and cDNA synthesis for molecular assessment 

Total RNA was extracted from follicles in each studied group (n = 12 in 3 replicates) using a TRIzol reagent extraction method (Invitrogen, Paisley, UK). By spectrophotometer, the quality of extracted RNA and its level were analyzed. The list of applied primers for *PCNA,* *FSHR,* and *β -actin* was summarized in table I.

### Real-time reverse transcription polymerase chain reaction (RT-PCR)

After extraction of total RNA and cDNA synthesis, according to QuantiTect SYBR Green RT-PCR kit (Applied Biosystems, UK), real-time RT-PCR was performed. Thermal conditions for the process included 3 steps; holding stage (95ºC for 5 min), step 2 was performed at 95ºC for 15 s, 58ºC for 30 s, and 72ºC for 30 s, and the last step (melt curve step) was continued at 95ºC for 15 s, 60ºC for 1 min, and 95ºC for 15 s. Then, the relative quantification of target genes to housekeeping genes was determined.

**Table 1:** The primers characteristics


**Target gene**	**Primer pair sequences**	**Accession number**	**Fragment size (bp)**
*** Β-Actin***	F: GGAAAAGAGCCTCAGGGCAT R: CTGCCTGACGGCCAGG	NM-007393	64
***PCNA***	F: 5AGGAGGCGGTAACCATAG3 R: 5ACTCTACAACAAGGGGCACATC3	NM-011045	76
***FSHR***	F: CCAGGCTGAGTCGTAGCATC R: GGCGGCAAACCTCTGAACT	NM-013523.3	79
PCNA: Proliferation cell nuclear antigen, FSHR: Follicle-stimulating hormone receptor			

### Ethical considerations 

The animals were used and cared for according to the Ethical Guidelines of Tarbiat Modares University. The Ethics Committee for Animal Research of the Tarbiat Modares University, Tehran, Iran, approved this study (Code: IR.TMU.REC.1394.272).

### Statistical analysis

Statistical analysis was conducted by SPSS 18 software (SPSS Inc., Chicago, USA). Quantitative variables were expressed as Mean ± SD. The survival rate, developmental rate, production of hormones, and results of real-time RT-PCR, were compared by one-way ANOVA, and Tukey's HSD was used as post hoc test. P < 0.05 was considered to be statistically significant.

## 3. Results

### The diameter of cultured isolated preantral follicles

**Figure 1:**
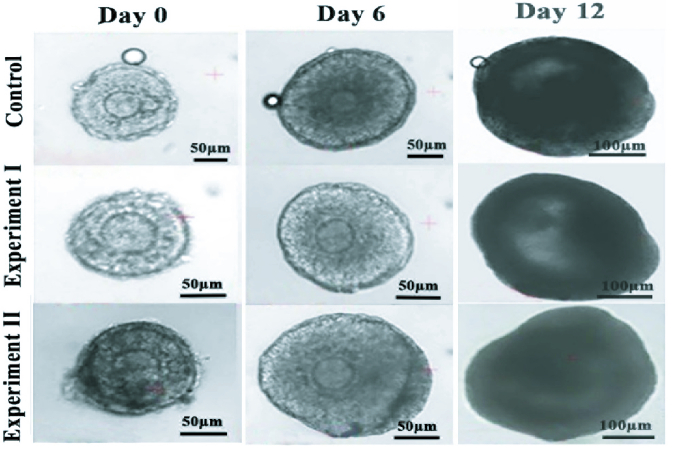
The inverted microscope images of 3-dimensional in vitro follicular development from day 0-12 in studied groups. Representative figures of cultured follicles in the control (first row), an experimental group I that was treated with sodium selenite (second row), and the experimental group II that treated with ovarian tissue extract (third row).

The morphology of cultured follicles in 3 studied groups under the inverted microscope is demonstrated in figure 1. During the culture period, the follicular size showed a remarkable increase, and their diameter changes during the culture period are summarized and compared in table II. No significant difference was observed in follicular diameters in all studied groups at the beginning of the culture. Moreover, on day 12 of the culture period, these ranges were significantly increased in all groups, and the mean diameter of culture follicles in the 2 experimental groups was significantly higher in comparison with the control group (p = 0.032). However, this mentioned parameter was higher in the SS-treated group (p = 0.019).

### Follicular developmental rate

The survival and antrum formation rates in follicles and the rate of released MII oocytes are summarized in table III. At the end of the culture period, amongst the studied follicles in control, SS, and OTE-supplemented groups, the survival rates were found to be higher in SS supplemented group (p = 0.032). In the control group the percentages of antrum formation and the rate of released MII oocytes were significantly lower than in other studied groups (p = 0.027); but there was no statistical difference between SS and OTE groups.

### Hormonal assay

The levels of estradiol and progesterone in the group that was treated with SS and those treated with OTE were increased (p = 0.021) but there was no significant difference between these groups in this regard (Table IV).

### The expression of *PCNA* and *FSHR* genes

**Figure 2:**
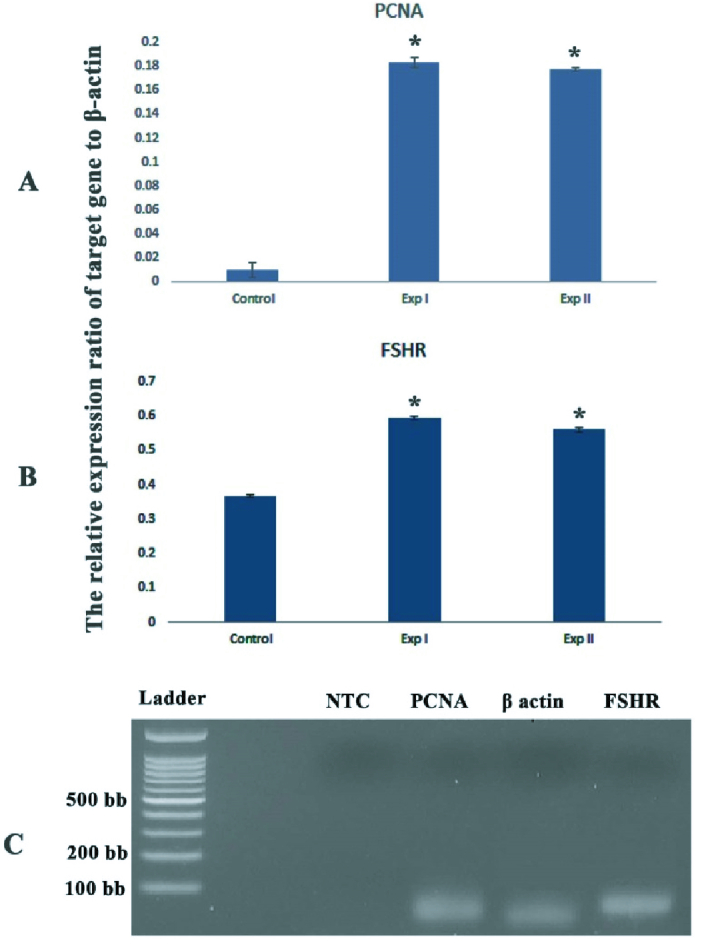
The comparison of gene expression in studied groups. The ratio expression of *PCNA* (A) and *FSHR* (B). The asterisk (*) shows a significant difference with the control group (p = 0.023). There was no significant difference between the 2 experimental groups (Exp I and Exp II). Values are presented as Mean ± SD. (C) Gel electrophoresis of PCR product in studied groups. NTC: Non-template control.

The mRNA levels of *PCNA* and *FSHR* genes were evaluated by real-time RT-PCR. The expression ratio of these target genes relative to the housekeeping (* β -actin*) gene and the representative figure of PCR product electrophoresis are shown in figures 2A and B. This figure shows the expression of these studied genes was lower in the control group in comparison with both experimental groups (p = 0.023). Also, the representative figure of gel electrophoresis of PCR products in studied groups was demonstrated in figure 2C.

**Table 2:** The preantral follicles' diameters (µm) during the culture period in the studied groups


**Groups**	**Day 0**	**Day 6**	**Day 12**
**Control**	152.9 ± 8.45	241.1 ± 7.50	342.05 ± 8.06
**SS treated (Exp I)**	152.75 ± 4.67	278.45 ± 8.82*	403.8 ± 11.3*
**OTE treated (Exp II)**	152.8 ± 4.16	271.6 ± 13.16*	383.97 ± 15.78*
Data presented as Mean ± SD. One-way ANOVA and Tukey's HSD. (*) shows a significant difference with the control group (p = 0.032). There was no significant difference between the 2 experimental groups (Exp I and Exp II). SS: Sodium selenite, OTE: Ovarian tissue extract			

**Table 3:** Developmental rates of in vitro culture of isolated preantral follicles


**Groups**	**Number of follicles**	**Number of survived follicle**	**Number of antrum formation**	**Number of GV oocyte**	**Number of GVBD oocyte**	**Number of MII oocyte**
**Control**	90	62 (69.38 ± 2.88)	41 (45.26 ± 3.64)	22 (5.54 ± 4.19)	27 (43.78 ± 3.53)	15 (24.66 ± 2.60)
**SS treated**	68	57 (84.58 ± 4.16) ${}^{\text{a}}$	30 (52.95 ± 2.28) ${}^{\text{a}*}$	17 (30.07 ± 5)	20 (35.06 ± 4.66)	20 (34.86 ± 1.98) ${}^{\text{a}**}$
**OTE treated**	90	68 (75.63 ± 2.17) ${}^{\text{b}}$	35 (51.21 ± 3.78)	21 (30.87 ± 4.89)	25 (36.74 ± 3.88)	22 (33.37 ± 1.11) ${}^{\text{a}**}$
Data presented as n (Mean ± SD). One-way ANOVA and Tukey's HSD. (a): Shows a significant difference with the control group in the same column (p = 0.023). (b): Shows a significant difference with the SS-treated group in the same column (p = 0.023). a* shows significant difference with the control group in the same column (p = 0.027). a** shows significant difference with the control group in the same column (p = 0.019). SS: Sodium selenite, OTE: Ovarian tissue extract, GV: Germinal vesicle, GVBD: Germinal vesicle breakdown, MII: Metaphase II						

**Table 4:** The levels of 17-β-estradiol and progesterone in collected media on Day 12 of culture


**Groups**	**E2 (pg/ml)**	**P4 (ng/ml)**
**Control**	1897.33 ± 40.51	16.82 ± 5.34
**SS treated (Exp. I)**	2573.33 ± 40.04 ${}^{*}$	36.50 ± 5.31 ${}^{*}$
**OTE treated (Exp. II)**	2458.76 ± 27.91 ${}^{*}$	35.51 ± 4.39 ${}^{*}$
Data presented as Mean ± SD. One-way ANOVA and Tukey's HSD. (*): Shows significant differences with the control group in the same column (p = 0.021). There was no significant difference between experimental groups I and II. SS: Sodium selenite, OTE: Ovarian tissue extract		

## 4. Discussion

The present study aimed to evaluate the effect of OTE obtained from the adult mouse on preantral follicular growth, development, and gene expression. For better evaluation of this effect, in another set of experiment, in parallel, the preantral follicles were treated with SS and their development and pattern of gene expression were compared with another experimental and control group*.*

Our results showed the survival, growth, and developmental rates of cultured follicles in 2 experimental groups that were treated with SS or OTE were higher than the control group, and these results revealed that the positive effects of these components on the follicular development.

In agreement with our results, several reports demonstrated the positive effects of SS as an antioxidant, proliferative, and maturation factor that improved follicular development and oocyte maturation (4-10). It was reported that a culture medium supplemented with different concentrations of SS (5, 10, and 15 ng/ml SS) promoted mouse in vitro follicular growth and maturation in a 2-dimensional culture system. Moreover, they demonstrated that 10 ng/ml of SS had better effects on the growth and maturation of the follicle (4).

Another study showed that the activity of glutathione peroxidase of oocytes and the DNA integrity of cumulus cells were increased in the presence of SS during IVM of oocytes (6). Selenium as an integral part of the enzyme glutathione peroxidase improves the survival rate of the cells by the decrease in the level of ROS (5).

In the present study, our results also confirm the similar effects of SS for enhancing follicular development in the 3D culture system. These results may be related to the antioxidant effects of SS by reducing the ROS or by reducing cell apoptosis (5, 7, 15-18).

According to the results of another part of this study, it is suggested that the positive effects of OTE to improve follicular and oocyte development may be related to components within this extract, such as several types of growth factors that could enhance the follicular diameters by increasing the number of granulosa and theca cells (11, 12). These factors are directly and indirectly involved in follicular growth and development. An increase in the number of follicular cells resulted in progress in follicular size that was demonstrated in both experimental groups during the culture period. It is suggested that SS as a proliferative factor and an antioxidant reagent enhances follicular development, while the OTE based on its component that mimics the natural condition and ovarian microenvironment showed the same effect.

However, this effect is more prominent in SS-supplemented group. One explanation for these results is that the presence of some additional components in OTE that are remained or produced during its preparation, interferes with the follicular development. Moreover, in the present study, we did not assay and analyze the content of OTE based on some limitations, thus more additional studies are needed to prove the mentioned suggestions.

The functional potential of granulosa and theca cells can be evaluated by measuring estradiol and progesterone production, and our observations showed that the levels of these hormones were increased in the culture media in both experimental groups in comparison with the control group. However, it was demonstrated that SS by stimulation of estradiol production could support in vitro and in vivo folliculogenesis (19). Also, it is suggested that the similar effect of OTE produces estradiol and progesterone by granulosa and theca cells; however, further study is needed to prove this suggestion.

Moreover, the molecular analysis in our study confirmed this suggestion and in this part of the study, we have evaluated the expression of 2 maturation markers in cultured follicles including *PCNA* and *FSHR* genes. As the follicular development proceeded, the expression and production of these proteins was increased and could be a good indicator of follicular growth (20-22). However, in the present study, our results showed the treatment of follicles in vitro by SS and OTE led to increased expression of *PCNA,* and* FSHR* genes. This observation confirmed again the beneficial effects of SS and OTE on the in vitro maturation of follicles.

A similar effect of SS was reported in an in vivo study by investigating the effects of SS on radiation-induced rat ovaries, which showed *PCNA* expression markedly enhanced in the irradiated rats that were treated with SS in comparison with the non-treated once (23). From the other point of view, FSHR is a G-protein coupled with 7 transmembrane domain receptors and is expressed in the granulosa cells (24). Moreover, the nuclear and cytoplasmic maturation of oocytes could be controlled by the content of OTE via the expression of maturation genes such as *FSHR*. FSHR is necessary for follicular development and is also responsible for producing androgens (25). It has been reported that when the transition from the primordial to primary follicle occurred, FSHR expresses progressively (26). The presence of FSHR in the early follicular stages has an indirect effect on follicular development via paracrine factors released by ovarian stromal cells (24, 27).

## 5. Conclusion

The OTE and SS have a positive effect on in vitro growth and maturation of mouse preantral follicles via over-expression of *FSHR* and *PCNA* genes.

##  Conflict of Interest 

The authors declare that there is no conflict of interest.
